# Quantitative models of nitrogen-fixing organisms

**DOI:** 10.1016/j.csbj.2020.11.022

**Published:** 2020-11-21

**Authors:** Keisuke Inomura, Curtis Deutsch, Takako Masuda, Ondřej Prášil, Michael J. Follows

**Affiliations:** a School of Oceanography, University of Washington, Seattle, WA, USA; b Institute of Microbiology, The Czech Academy of Sciences, Opatovický mlýn, Třeboň, Czech Republic; c Department of Earth, Atmospheric and Planetary Sciences, Massachusetts Institute of Technology, Cambridge, MA, USA

**Keywords:** Nitrogen fixation, Nitrogen fixers, Quantitative model, Mathematical model, Photosynthesis, Oxygen

## Abstract

Nitrogen-fixing organisms are of importance to the environment, providing bioavailable nitrogen to the biosphere. Quantitative models have been used to complement the laboratory experiments and *in situ* measurements, where such evaluations are difficult or costly. Here, we review the current state of the quantitative modeling of nitrogen-fixing organisms and ways to enhance the bridge between theoretical and empirical studies.

## Graphical abstract

**Figure:**
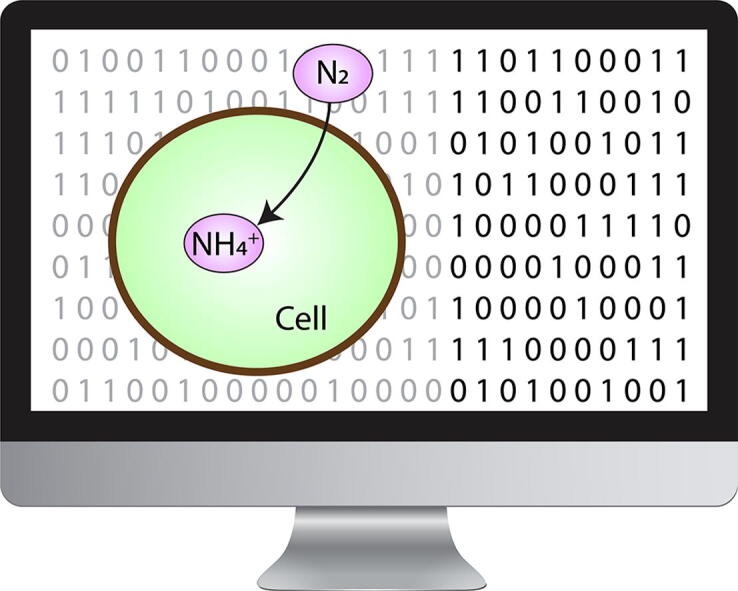

## 1 Introduction

### 1.1 Nitrogen fixation and its influence in the environment

**Fig. 1:**
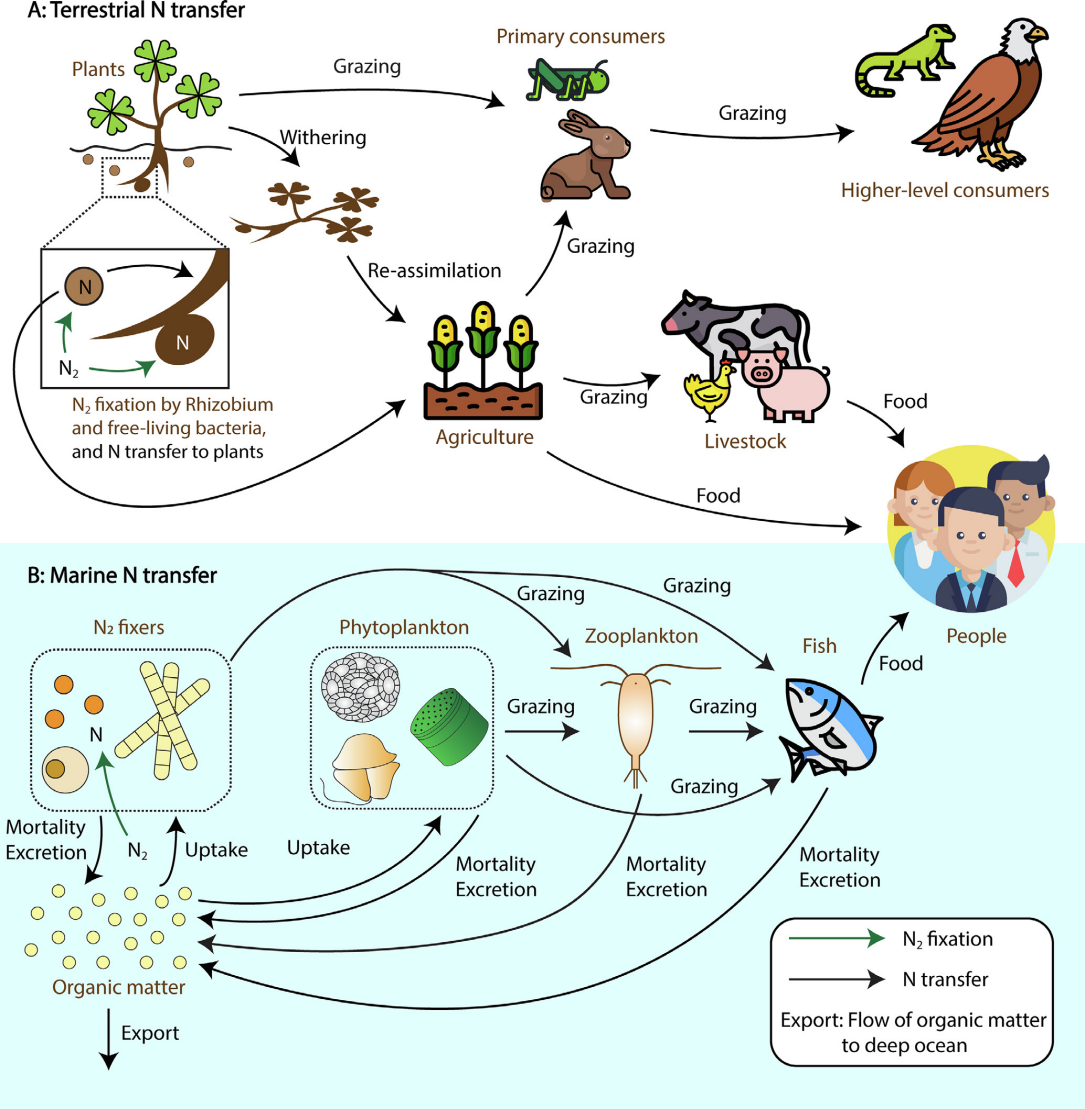
N flows in (A) terrestrial and (B) marine systems. “N” indicates fixed N whereas “N_2_” indicates dinitrogen gas.

Biological nitrogen fixation (hereafter “N_2_ fixation”) is the dominant source of reactive nitrogen (N) in the Earth system, far exceeding abiotic sources from lightning [1], [2], [3], [4]. It provides bioavailable N to the biosphere supporting organismal growth of various trophic levels and human lives (Fig. 1). On land, bioavailable N (fixed by e.g., *Rhizobium* [5], [6], [7], [8] and free-living bacteria [4], [7], [8], [9]) is transferred to the primary producers (e.g., plants, cyanobacteria), which are then transferred to consumers. N_2_ fixation is of special interest in agricultural sectors [7], [8], [9], [10], since it is an environmentally sustainable source of bioavailable N, reducing the use of fertilizer, which is economically and environmentally costly [8], [9], [10].

In the ocean, the majority of N_2_ fixation is performed by prokaryotic phytoplankton, which is then consumed by larger plankton and by fish, some of which are consumed by human beings (Fig. 1). The fixed N released (often combined with C) from these organisms is a component of ecosystem N inputs [11], [12]. It has been estimated that about a half of fixed, or bioavailable N, originates from microbial N_2_ fixation, important also for the coupled the C cycle [1], [13]. A greater oceanic inventory of fixed N may increase the primary production [11], [14], [15] and export of organic C to the deep ocean [11], [14].

### 1.2 Key controls for N_2_ fixation and their management at a cellular level

Although N_2_ fixation has an influence at the ecosystem scale, the rate of N_2_ fixation is constrained at a cellular level. In this section we explore major limiting factors (i.e. reduced C, inorganic nutrients and O_2_) and how the cells acquire and manage them. These are the key factors in the development of the models for N_2_ fixing organisms (hereafter N_2_ fixers).

#### 1.2.1 Reduced C

N_2_ fixation requires electrons and energy:

(1) $$N_{2}+{8e}^{-}+{10H}^{+}+16ATP+16H_{2}O\to 2NH_{4}^{+}+H_{2}+16ADP+16P_{i}$$

Reduced C, such as carbohydrates and lipids, provides the electrons and energy for N_2_ fixation, thus influencing the rate of N_2_ fixation, especially when C is limited and/or other nutrients are abundant. Organic carbon is oxidized by metabolic processes (e.g., TCA cycle), providing reducing agents (e.g., NADH) [16], [17], [18], [19], which are used to transfer electrons to nitrogenase [20], [21], [22]. Such reducing equivalents donate electrons to the electron transport chain and ATP synthesis [16], [17], the energy carrier for stepwise reduction of N_2_ to ammonia (NH_3_) [23], [24], most of which is instantly converted to ammonium (NH_4_^+^) at typical intracellular cellular pH.

**Fig. 2:**
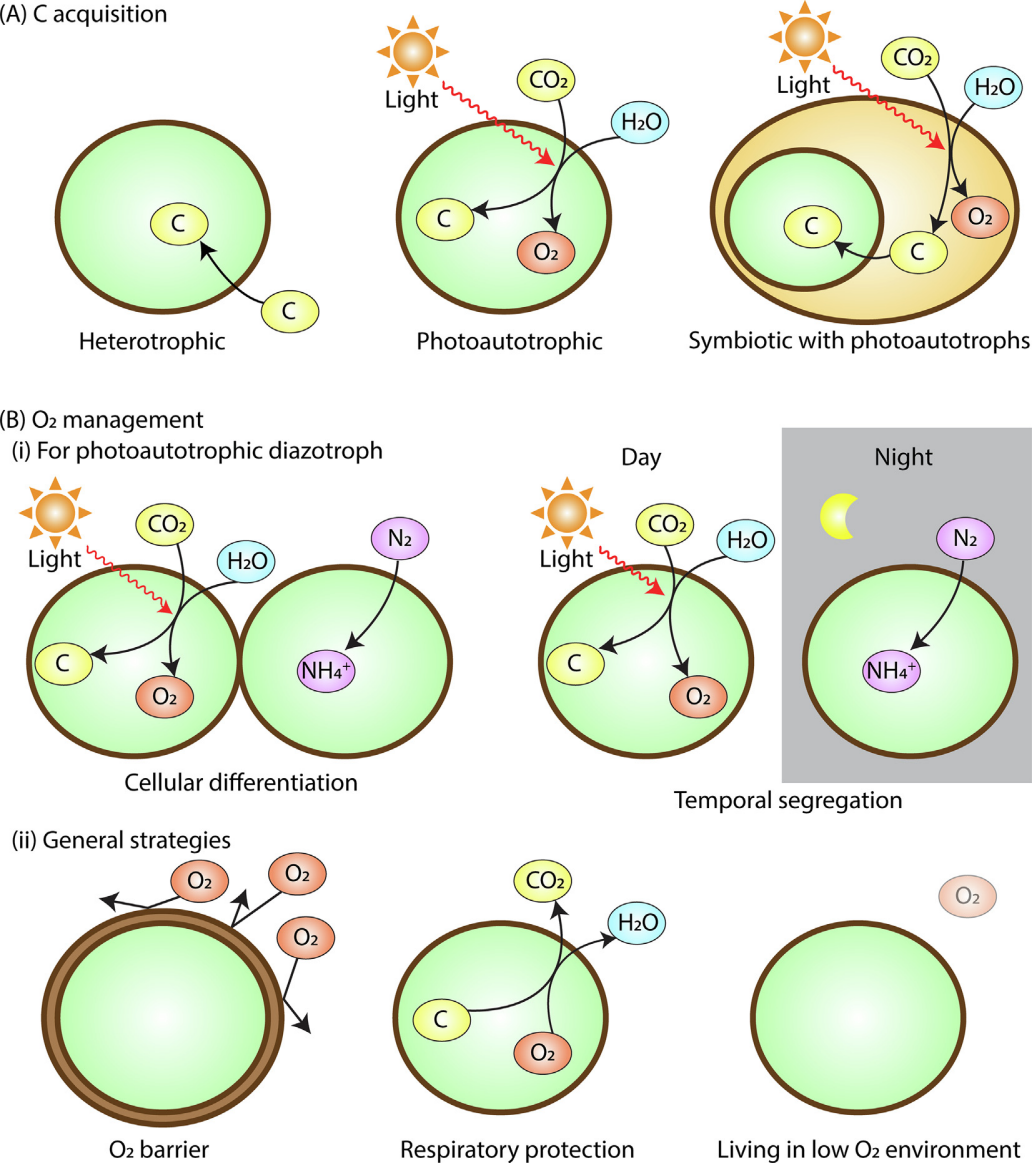
Strategies for (A) Biomass (organic) C acquisition and (B) O_2_ management. Here “C” in a yellow oval represents biomass C. The following are example organisms: (A) Heterotrophic: *Azotobacter*, *Clostridium*. Photoautotrophic: *Crocosphaera*, *Trichodesmium*, *Anabaena*. Symbiotic: *Rhizobium*, UCYN-A. (B) Cellular differentiation: *Anabaena*, *Richelia*. Temporal Segregation: *Crocosphaera*, *Cyanothece*. O_2_ barrier: *Azotobacter* (proposed [72], predicted [53] and supported [81], [82]), *Crocosphaera* (predicted [53], [75]), *Anabaena*, *Trichodesmium* (predicted [83], [84]). Respiratory protection: *Azotobacter*, *Crocosphaera* (predicted [75], [85]), *Trichodesmium* (predicted [83]). Living in low O_2_ environment, *Clostridium*. (For interpretation of the references to colour in this figure legend, the reader is referred to the web version of this article.)

There are three main ways to acquire organic C (Fig. 2A). One is from the external environment (heterotrophic C acquisition), which is common in soil [9] and sediments [25], but recognized in the open ocean as well [26]. In this case, the availability of organic C limits the rate of N_2_ fixation [27]. The second way is through photosynthesis, in which light energy is used to separate electrons from water, which in turn is used for reducing CO_2_ [16], [17], [18]. In this way, the cells can access a ubiquitous source of C but light availability is essential and thus the process is limited to the day time in the surface ocean. The third way is through symbiosis with photoautotrophic organisms, such as plants and phytoplankton [28], [29], [30], [31], [32]. The photoautotrophic hosts provide C to the N_2_ fixer, and in return, the N_2_ fixers provide fixed N to the host.

#### 1.2.2 Phosphorus and iron

Phosphorus (P) and iron (Fe) are also important for N_2_ fixation [33], [34], [35], [36], [37], [38]. Fe is an essential trace metal for N_2_ fixation as it forms co-factors for nitrogenase (nitrogen-fixing enzyme) [23], [24]. P, on the other hand, influences the rate of N_2_ fixation rather indirectly, as it is used for various parts of the cells that holds nitrogenase, such as cell membrane, ATP (energy transferring molecule), DNA and RNA [16], [17], [18], [19]. We note that nitrogenase requires other trace metals such as molybdenum (Mo) and vanadium (V) [24], [39], [40], [41], [42]. In this review, we focus on Fe, since it has been more explicitly represented in quantitative models.

Inorganic forms of these nutrients are transported into the cell by transporters [43], [44], [45], since these molecules are generally charged in water (e.g., PO_4_^3−^, Fe^2+^) and do not usually go through cell membrane. Cells have various strategies for acquiring these, such as the use of high affinity transporters for PO_4_^3−^ [43], [46] and physical attachment to Fe rich particles [47]. Some cells live within other microbial cells or are symbiotic to plants [28], [29], [30], [31], [32], potentially acquiring these molecules from the hosts. We note that organic P [43], [46], [48] and Fe associated with organic molecules [49], [50], [51], [52] can also be used by N_2_ fixers.

#### 1.2.3 O_2_

O_2_ is essential for respiration but is rather detrimental for N_2_ fixation [53], [54], [55]. Especially, under normal aquatic O_2_ concentrations, the Fe protein in nitrogenase complex loses its activity irreversibly [54]. Thus, N_2_ fixing cells must create a low oxygen environment in the cytoplasm, where nitrogenase exists, to enable N_2_ fixation. This is particularly challenging for photosynthetic N_2_ fixers since photosynthesis produces O_2_ [16], [17], [18], [19]. One simple way to avoid it is to fix N_2_ during the night [56], [57], [58], [59] (Fig. 2B). Because photosynthesis requires light and only occurs during the day, the dark period is an ideal time for N_2_ fixation. However, this strategy is not universal; some photoautotrophic organisms fix N_2_ during the day (e.g., *Trichodesmium* and *Anabaena*) [60], [61], [62], [63]. Some of these organisms (e.g., *Anabaena*) form filaments and have differentiated cells (heterocysts) for N_2_ fixation [64], [65], segregating the sites of photosynthesis and N_2_ fixation.

Although these strategies are effective in managing photosynthetically originated O_2_, they may not be sufficient, since the non-polar O_2_ molecules can diffuse into the cell from the external environment [66], [67]. O_2_ in the environment is often high (e.g., generally > 150 µM in the surface ocean [68], [69], [70] and nearly saturated (~20% O_2_) in the shallow layers of soil [71]), which creates gradient of O_2_ concentration that favors O_2_ flows from the external environment into the cell (Fick’s first law of diffusion).

One way that organisms manage this problem is to create a barrier around the cytoplasm (Fig. 2B) [64], [72], [73]. Such a barrier would minimize the O_2_ diffusion and allow the cells to keep the steep gradient of O_2_ between the cytoplasm and external environment. However, an excessive barrier could also limit the diffusive source of N_2_. Another way to manage O_2_ is respiratory protection (i.e. respiration to reduce intracellular O_2_) [53], [74]. Even if there is a high O_2_ flux into the cell, if the rate of respiration matches the flux, a low intracellular O_2_ can be maintained [27], [53], [75]. Finally, there are organisms that live in low O_2_ environments such as in sediments [25], [76], [77] and Oxygen Minimum Zones in water columns (OMZs) [78], circumventing the O_2_ problem. Some symbiotic systems may provide local environments with low O_2_ [79], [80]. The threshold of environmental O_2_ below which N_2_ fixation occurs depends on the potential level of respiration and other O_2_ management mechanisms (such as O_2_ barrier) [53].

### 1.3 Quantitative modeling of N_2_ fixers

To quantify the activities of N_2_ fixers and the effect of the factors controlling N_2_ fixation, extensive measurements have been conducted in the open ocean [86], [87], [88] and on land [10], [89], [90]. To study the physiology of N_2_ fixers, a significant number of experiments and *in situ* observation have also been conducted [9], [91], [92]. However, there are still significant unknowns and experiments/observations are generally costly and many properties are difficult to measure: even major methods for measuring the rate of N_2_ fixation have been questioned [93], [94], [95], [96], [97] and it is still challenging to directly measure the intracellular concentration of O_2_, which is detrimental to nitrogenase, the N_2_ fixing enzyme complex [53], [54].

**Fig. 3:**
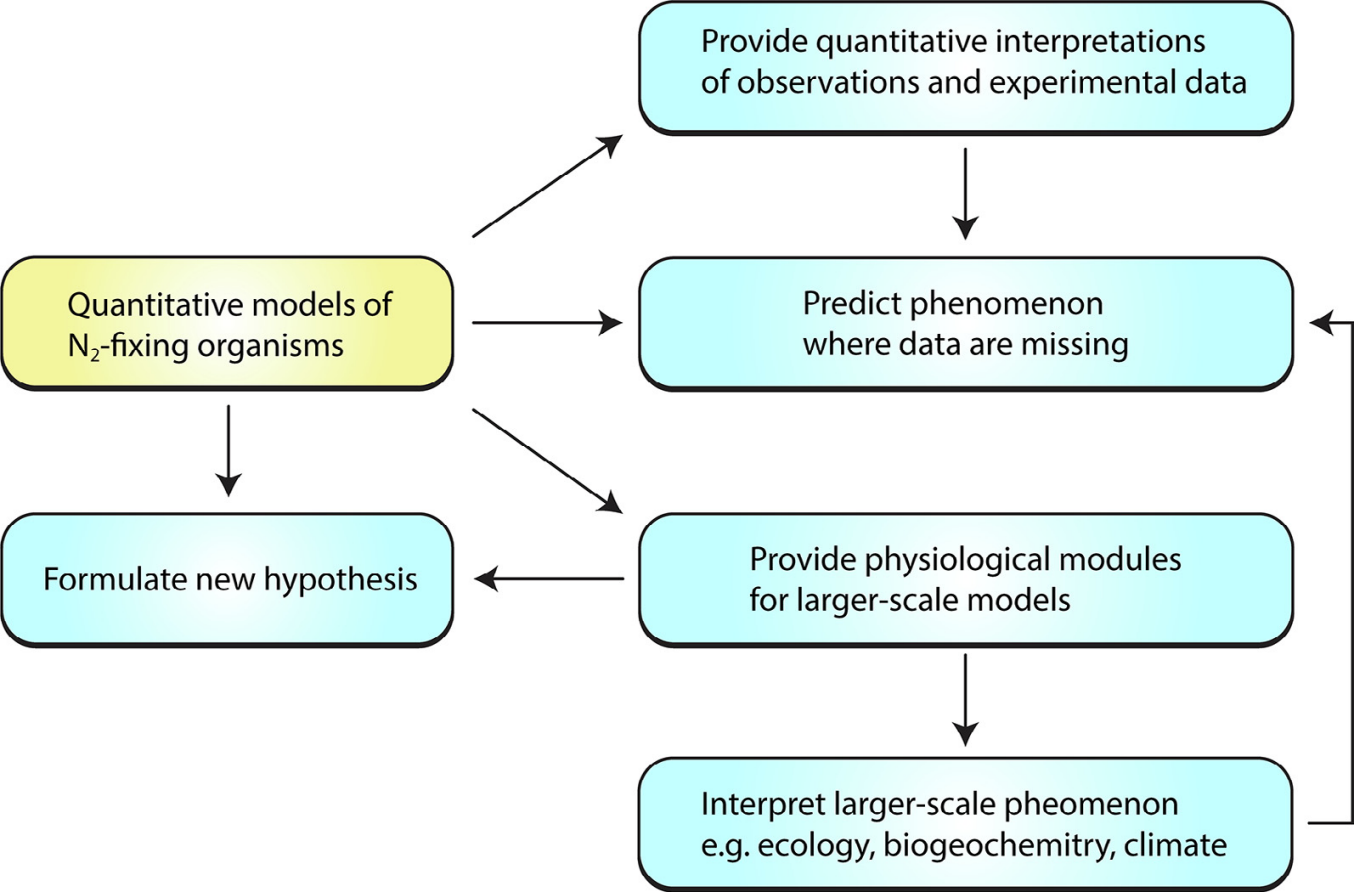
Roles of quantitative models of N_2_ fixers. Arrows indicate causes and effects.

Quantitative models (see Table 1 for the definition) have been used to complement biological measurements, providing mathematical theories to interpret observations, formulate new hypotheses, and make predictions where data are missing (Fig. 3). For example, based on the model of simple cellular metabolisms as well as the available environmental factors (such as nutrient, light and temperature), models may predict the rate of N_2_ fixation as well as intracellular concentration of O_2_ as well as the fate of intracellular C or cellular growth [27], [53], [83], [98], [99], [100]. Such models of N_2_ fixers can be used to quantitatively interpret experimental data (e.g., what controls the growth or N_2_ fixation rates of cells at a certain time point or under a certain condition?). They can also be implemented in larger-scale ecosystem simulations, such as terrestrial [101], [102], [103] and regional [104], [105] and global [106], [107] ocean models, which are used for interpreting *in situ* observations of biogeography and N_2_ fixation rates [88], [106], [108], [109], [110] and for predicting changes in global ecosystems (such as plankton competitions and food transfers) [104], [106], biogeochemical cycles (such as N, C, and trace metal cycles) [104], [107], [111], [112], and climate [113], [114], [115], [116], [117].

**Table 1:** Some modeling terms and definitions in this paper.

Name	Definition
Quantitative model	A mathematical description combined with quantification of a phenomenon, often solved by computers. In this paper, we simply use a term “model” for such a model. The antonym for this term is “qualitative model”, which describes phenomenon without numerical evaluation. In this paper we focus on quantitative approaches.
	
Biogeochemical model	A mathematical description or simulation of biologically, chemically and physically mediated elemental and chemical fluxes in the environment. Typically focused on ecosystem and global scales, and relationships with the Earth’s environment. In global-scale biogeochemical simulations, biological growth and activities are generally highly simplified and often implicit.
	
Ecological/Ecosystem model	A model that simulates the growth and activities of biological organisms (generally two or more) in a particular environment (from regional to global scales).
	
Cellular/Physiological/Metabolic model	A model that simulates the metabolism of microbial cells, resolving fluxes and sometimes reservoirs of molecules within the cell.
	
Optimization model	A model in which parameters are tuned systematically in order to best match observed states or to fulfill certain conditions, such as maximization of a certain output (e.g., biomass production).

Slash “/” in the name indicates that we use these terms interchangeably.

## 2 Type of model

**Fig. 4:**
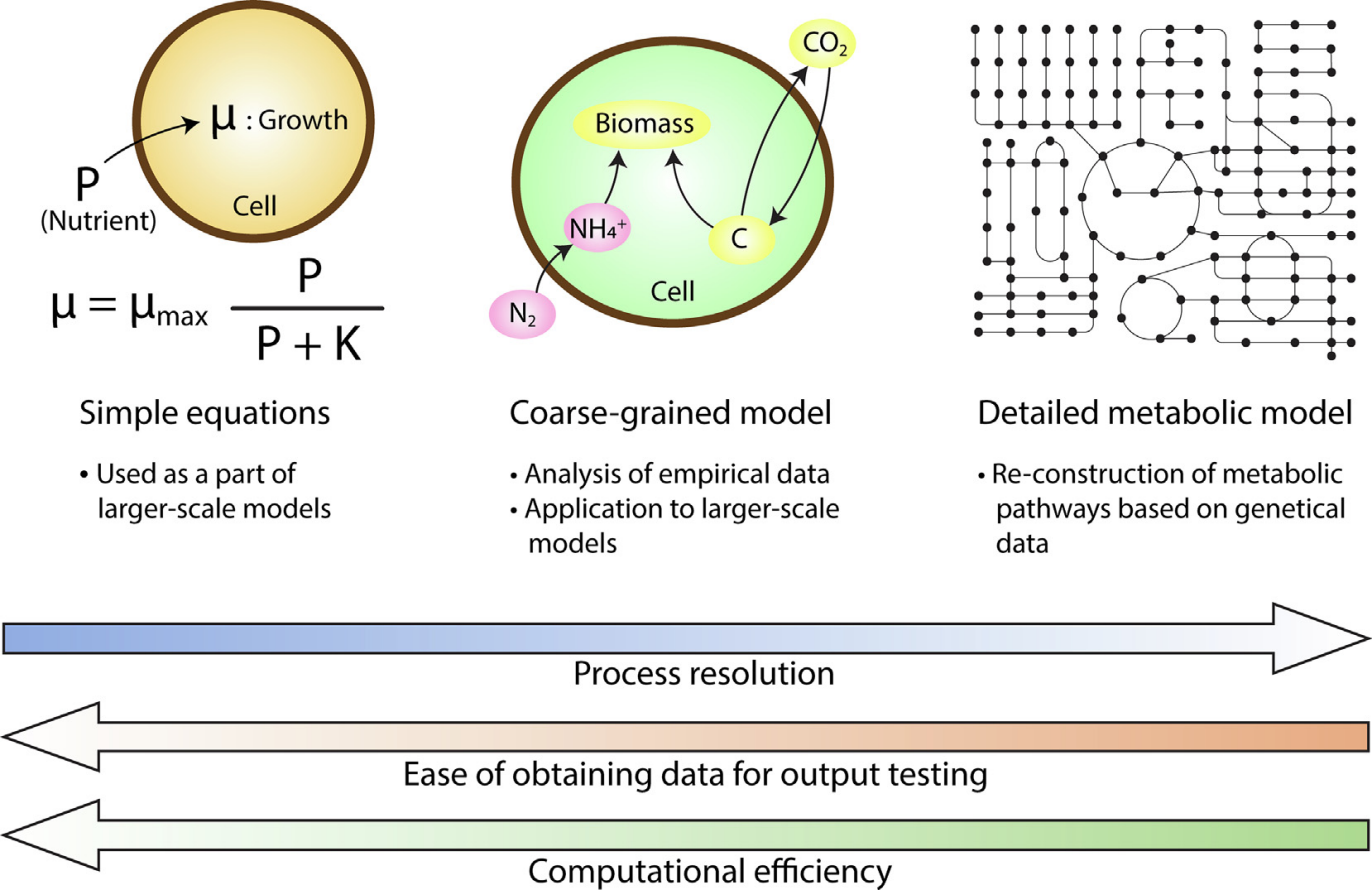
Schematics of three different types of models. *μ*_max_: maximum growth rate. K: half saturation constant for growth based on nutrient concentration following Monod kinetics [118], widely used in ecosystem modeling [102], [104], [107], [124]. Examples of coarse-grained model and detailed metabolic model include Cell Flux Model (CFM) [53], [75], [83], [121]. One widely used detailed metabolic model is Flux Balance Analysis (FBA) [135], [136], [137], [138].

A number of models have been developed to express physiology of N_2_ fixers, but they can broadly fit into one of the three groups: simple equations (analytical theory with relatively small number of equations and variables), coarse-grained models, and detailed metabolic models (Fig. 4). The resolution of metabolic processes increases in this order, but computation becomes less efficient (i.e. taking longer time for the same amount of computational power) and model-data comparison becomes harder. These three types of models are complementary to each other and are used for different purposes. We describe each type with examples in the following part.

### 2.1 Simple equations

The simplest category of models describes populations and rates with only a few equations, often used as a part of the ecological models. Good examples are Monod-type (Michaelis-Menten like saturating relationship) equations [118] used in ecosystem models (see Table 1 for the definition) [104], [106], [119], where the growth rate is described as a simple function of external environmental factors, such as light, temperature and nutrients. The rate of N_2_ fixation can be calculated based on the growth and elemental stoichiometry of the cells. Specifically, these models compute N_2_ fixation by multiplying the growth rate, biomass N per cell, and cell population such that N_2_ fixation is implicitly sufficient to meet nitrogen demand. In such models, intracellular properties, such as elemental stoichiometry of cells and macromolecular allocations, are generally assumed constant, despite the fact that in reality they generally vary significantly [120], [121], [122], [123].

Despite their simplicity, simple equations are the main way to express physiology of N_2_ fixers in large-scale models, such as ocean ecosystem models [104], [106], [119], [124]. One key reason is computational efficiency; more complex biological descriptions require more state-variables and more computational operations, thus increasing both memory and processing demands which can become prohibitively expensive. Although highly idealized, these ecosystem models with simple equations seem to broadly capture the observations [104], [106], [110], [125]. Here, it is assumed that the growth rates of N_2_ fixers are not limited by N but by P and Fe, allowing them to acquire a niche where N is scarce. In general, the effects of the “end product suppression” by fixed N are not considered, despite its potential importance. Using the simplified equations, we can connect to ecological theory for the shaping of communities: under steady state conditions the simplified equations lead to a resource supply ratio theory, suggesting that the niches of N_2_ fixers are constrained based on the ratio of nutrient sources (specifically N, P, Fe) [34], [126].

Idealized mathematical descriptions (simple equations) are also developed and employed for terrestrial simulations. Some models simply assume that the rate of N_2_ fixation is proportional to the amount of biomass [103], [127], [128], [129]. Other models assume that the rate of N_2_ fixation is a function of temperature [101], [130]. Similar to ocean models, Michaelis-Menten type equations are often used, where the rate of N_2_ fixation is calculated based on the available C and N [102]. It is noteworthy that most models are formulated in the context of symbiosis with plants [102], [103], [127], [128] due to the existence of wide-spread plants-*Rhizobium* symbiosis. In the context of symbiosis, some terrestrial models relate net primary production [89], [131], [132] or evapotranspiration [89], [133] of plants to the rate of N_2_ fixation. The net primary production of the host plant has been modeled based on the cost for N_2_ fixation and light availability [134]. Whereas most models are developed in the context of symbiosis, there are models that combine both symbiotic and non-symbiotic N_2_ fixation, prescribing different temperature functions to each type [101], [130].

Simple models have the advantage of mathematical transparency; they are easier to interpret and apply. They are also computationally cheap for global-scale biogeochemical applications. On the other hand, they may gloss over many processes which are known to be important and they are usually not easy to calibrate or test with the exploding database of ‘omics observations because the currencies of simple models tend not to translate simply into genes or transcripts. For example, gene-copy per cell is highly variable taxonomically, thus hard to relate to biomass. Transcription can be fleeting and highly taxonomically specific. One way to exploit ‘omics data more directly is to develop models at the genome-scale.

### 2.2 Detailed metabolic models

Detailed metabolic models are on the other side of the complexity spectrum, since they include genome-scale simulations which represent metabolic networks of hundreds of reactions (Fig. 4), generally using FBA (Flux Balance Analysis) [135], [136], [137], [138]. FBA is a mathematical method for simulating a balanced metabolic flux network of any size based on optimization of fluxes, which is done by matrix computation. Many potentially viable network configurations are possible in order to satisfy given boundary conditions and optimization targets. Optimal network configurations are sought by maximizing biomass production [137], [138], minimizing a number of metabolic pathways [139], [140] or other constraints. The strength and a key application of such simulations is to predict metabolic organization and fluxes from observed genomes [135], [141], [142]. The volume of genome sequences is rapidly increasing, enabling the application of FBA to a wide range of organisms including N_2_ fixers.

Despite the wide use of FBA, there are still challenges. First, the model output is often hard to compare with data. It is rarely the case that data to constrain hundreds of pathways are available [143], and the comprehensive test of the output is challenging and often highly qualitative. The models typically evaluate metabolic fluxes but not the abundance of metabolites or macro-molecules, which have been actively measured recently ([123], [144], [145], [146]). Genome scale simulations may be computationally demanding in order to find the optimum (see Table 1 for definition) of thousands of solutions [135], [138]. Although a genome-scale FBA can be run on a laptop computer, current codes can take seconds to minutes for a single solution, limiting their application in large-scale ecosystem simulations. However, there have been efforts to overcome this challenge (e.g., [147], [148], [149]).

### 2.3 Coarse-grained models

Coarse-grained models lie between the complexity of the simplified equation and genome-scale FBA approaches described above: they include more detailed physiologies than simple analytical equations may allow, but resolve fewer metabolic pathways than the genome-scale simulations [150] (Fig. 4). Typically they resolve an idealized and simplified representation of metabolic pathways at the level of major cellular function including biosynthesis, respiration and photosynthesis as well as N_2_ fixation as a whole [53], [98], [99], [121], [151]. These models are typically constrained by conservation constraints on elemental, electron and energy budgets [27], [53], [152], [153]. Some coarse-grained models resolve macromolecular allocation [121], [122], [154], which can be compared with emerging sources of macromolecular and proteomics data.

Whereas there are variations in coarse-grained models, they can be made computationally efficient and possibly incorporated into larger models. Especially, optimization related loops within the computational codes are not essential [75], [83], [121], which would increase the computational load significantly. The implementation of a coarse-grained model of N_2_ fixer in regional-scale model has been recently done for a major marine N_2_ fixer, *Trichodesmium* [105]. The implementation of coarse-grained models of N_2_ fixers in global scale models has not been done, but is possible. Although comprehensive metabolic pathways may not be reconstructed from genomic data as can be done for FBA, metabolic pathways can be selectively included [155], creating variations in the network of metabolic fluxes [27], [75], [153], [156]. Compared to other two types of models, coarse-grained models do not have a set of “standard formulas” and can be flexibly modified for specific purposes or available data: especially suited for bulk measurements such as those from batch-cultures or chemostat-cultures [58], [85], [123], [146], [157], [158], [159].

## 3 Modeled organisms

**Fig. 5:**
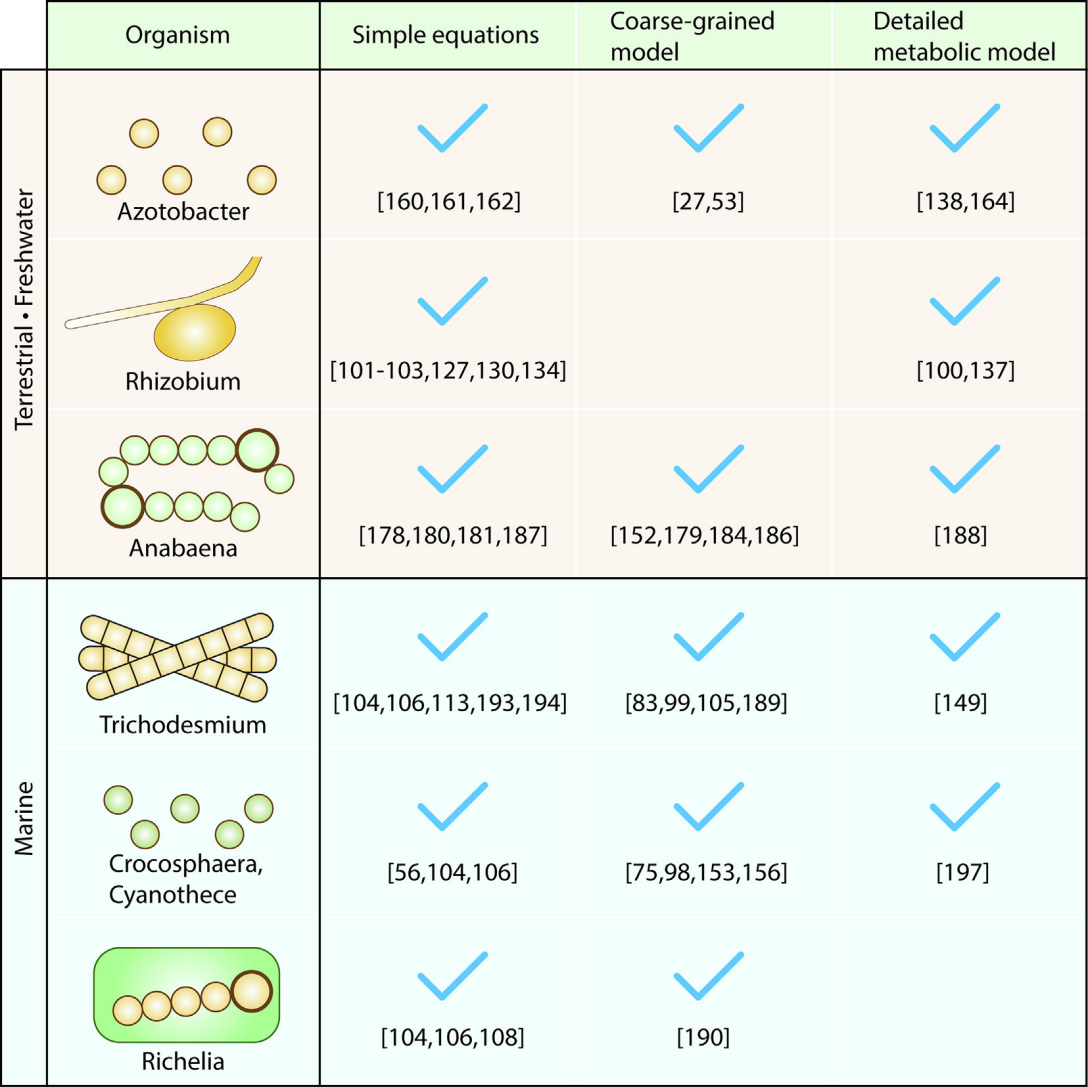
A list of major modeled N_2_ fixers and current state of model development. Checkmarks indicate that the model has been developed in each way. Numbers below the check marks are example references.

For obvious reasons, most physiological models have been developed around “model organisms” which have been extensively studied in laboratories. Here we discuss selected major model organisms and group them based on the environment (terrestrial/freshwater and marine), the modeling approaches applied, (Fig. 5) and the inferences gained from those models.

### 3.1 Nitrogen fixers in terrestrial and freshwater environments

Terrestrial N_2_ fixers are classified broadly based on whether heterotrophic or photoautotrophic and whether free-living or symbiotic (Fig. 5). Here we select key organisms for quantitative models and explore which modeling strategies have been applied.

#### 3.1.1 Azotobacter

Key modeled free-living organisms are soil dwelling heterotrophic unicellular bacteria (Fig. 5), *Azotobacter vinelandii*, which is also considered as “a model organism” in laboratory studies [9]. During the latter half of the 20th century, simple equations were used to describe the quantitative relationships between the growth rate, yield and maintenance costs as well as substrate concentration [160], [161]. Similarly, simple equations were applied to the chemostat culture data of relationships between resource C:N ratio and the rate of N_2_ fixation under various O_2_ concentrations [162], where different parameters are prescribed for each O_2_ concentration. Recently, a coarse-grained model (Cell Flux Model or CFM) has been developed [27], [53], which simulates these chemostat data sets [161], [162], [163] with a single-set of parameters. This model revealed a high C cost of respiratory protection (respiration for reducing intracellular O_2_ to protect nitrogenase, which is O_2_ sensitive) both under diazotrophic condition [53] and when NH_4_^+^ is added to the culture [27]. Even when N_2_ fixation did not occur due to the addition of NH_4_^+^, the respiratory protection occurs, suggesting that respiratory protection is decoupled from N_2_ fixation [27]. The study provided a quantitative baseline for modeling the direct and indirect costs of N_2_ fixation more generally. During the similar time period, FBA was applied to *Azotobacter* and showed that O_2_ availability affects TCA cycle, PP pathway and alginate and P3HB (poly-3-hydroxybutyrate) biosynthetic fluxes [164].

#### 3.1.2 Rhizobium

A major terrestrial symbiotic heterotrophic N_2_ fixer is *Rhizobium*, which creates bacteroids within the root nodules (legumes) of plants (e.g., clovers and alfalfa) [165] (Fig. 5). The bacteroid fixes N_2_, much of which is transported to the plants and supports their growth. Several models have been developed based on simple equations for various purposes. For example, simple equation models representing symbiotic N_2_ fixers in legumes [101], [102], [103], [127], [130], [134], have been used for various purposes including estimation of the magnitude of terrestrial N_2_ fixation.

As more genomics data for *Rhizobium* become available [166], [167], detailed metabolic models have also been developed. Recently FBA was applied to *Rhizobium* [137] and showed different metabolic regimes based on O_2_ and carbohydrate update rates. This FBA framework is further extended based on the genomics and proteomics data [100]. However, coarse-grained type models of these systems do not seem to exist, despite their potential benefits. This might be due to the difficulty in bulk quantitative measurements of bacteroid metabolism/properties as they are tightly integrated in plant tissues, which would be essential in constraining the model.

#### 3.1.3 Anabaena

*Anabaena* is a cyanobacterium (photo-autotrophic prokaryotic alga) both free living and symbiotic with fern plant (*Azolla*) [168], [169], [170]. We note that genus *Anabaena* has been renamed to *Dolichospermum* but here we use the term *Anabaena* as it has been more commonly used. They form a chain of cells (trichome) (Fig. 5), within which there are heterocysts [64], [171], [172]. Specifically, heterocysts are visually distinct with thick glycolipid layers on the cell membrane, which protects the cytoplasm and thus nitrogenase from O_2_ [65], [73], [173]. Some studies show that bacteria specifically associated with heterocysts can provide respiratory protection from O_2_ [174]. Heterocysts do not evolve O_2_ since it lacks functional photosystem II (PSII), which evolves O_2_, but can harvest light energy with photosystem I (PSI) [64], [65], [175]. The light energy harvested by PSI can be used for ATP synthesis based on the cyclic electron flow and proton pumping, possibly supporting N_2_ fixation [176]. Other cells, termed vegetative-cells, photosynthesize during the day, providing fixed C to heterocysts [177].

A simple equation model of *Anabaena* has been developed predicting the growth rate based on temperature, light and phosphorus availability and its intracellular quota [178]. Also, a coarse grained model of *Anabaena* has been developed, resolving the clock-controlled and non-clock-controlled protein synthesis, capturing the observed diurnal patterns of protein synthesis [179]. Later, these two models are combined, resolving heterocyst differentiation based on a wide range of laboratory experiments [152]. We note that there have been various modeling efforts to predict heterocyst development with various modeling complexities [180], [181], [182], [183], [184], [185], [186]. There also exist models of simplified equations for predicting growth rates [180], [187]. Furthermore, FBA has been applied to *Anabaena* resolving both vegetative cells and heterocysts [188], which suggests the importance of the exchange in metabolites in achieving observed growth rates.

### 3.2 Nitrogen fixers in marine environments

Although there is a wide variety of marine N_2_ fixers, currently most quantitatively modeled organisms are cyanobacteria (Fig. 5) [75], [83], [99], [153], [189], [190]. Since cyanobacteria produce O_2_ through photosynthesis, O_2_ management is one key topic in modeling studies and is chiefly considered with coarse-grained models due to their capability of quantifying intracellular molecules [75], [83], [191]. Here we explore three of the key N_2_ fixers in the ocean [2], [3] and their distinct O_2_ management strategies.

#### 3.2.1 Trichodesmium

*Trichodesmium* is a filamentous multicellular N_2_ fixer distributed across the ocean (Fig. 5) [2], [3]. They fix N_2_ during the day, when O_2_-producing photosynthesis occurs [60], [192]. The distribution of *Trichodesmium* has been predicted by various ecosystem models [104], [106], [193], [194] that express its physiology by simple equations directly connecting external environments to the rate of growth and N_2_ fixation. In such models, it is generally assumed that the uptake of fixed N is zero and the maximum growth rate is smaller than non-N_2_-fixing counterpart as a handicap for N_2_-fixing capability. *Trichodesmium* has also been modeled in a coarse-grained way, the beginning of which resolves the diurnal cycle of C and N, showing that N_2_ fixation increases when the availability of fixed N decreases [189]. More recently, a simplified version resolves intracellular O_2_ [83], predicting multiple O_2_ management mechanisms, such as respiratory protection and barrier against O_2_. An optimization based coarse-grained model resolving C, N and P fluxes has also been developed [99], and incorporated into regional marine ecological framework [105], showing that low P availability favors N_2_ fixation, which explains the presence of N_2_ fixation under high N:P supply ratios. There is also a model that resolves Fe allocation as well as C concentrating metabolism [195], predicting significant decrease in N_2_ fixation by *Trichodesmium* especially in Fe limited regions. Genome-scale FBA has been applied to *Trichodesmium* predicting that about 15% of cells are actively fixing nitrogen (diazotrophic), which is within the range of observation, and about 30% of total fixed N leaks to the environment [149].

#### 3.2.2 Crocosphaera

*Crocosphaera* is a unicellular cyanobacterium (Fig. 5) mainly found in oligotrophic oceans [2], [3], [196]. It fixes N_2_ during the dark [85], temporally avoiding O_2_ evolving photosynthesis [60]. A proteomics study highlighted the recycling of iron within the cell between nitrogenase and photosystems on a daily basis [56]. In ocean ecosystems, *Crocosphaera* has been included as simple equations (often represented as unicellular N_2_ fixers) [56], [104], [106]. One model illustrated the fitness advantage and extended range enabled by daily Fe recycling in the oligotrophic Pacific where Fe is scarce [56].

There are multiple types of coarse-grained models for *Crocosphaera*. Some resolve functional molecules without diurnal cellular cycles [153], [156]. One model resolves diurnal cycles of cellular C and N metabolisms, with more coarse molecular representation [98]. Recently, a model with a diurnal cycle resolving intracellular O_2_ concentrations and Fe cycles has been developed showing that O_2_ and the level of respiration are key factors in constraining their niche in warm waters (>20 °C) [75]. Furthermore, a model resolving heterogeneous N_2_ fixation among the population showed that such heterogeneity decreases the cost for O_2_ management and extends the depth niche of *Crocosphaera* [191].

FBA has been applied to a similar diazotrophic cyanobacteria *Cyanothece* strain ATCC 51142 [197], which is found in coastal waters [198] and has recently been re-classified as *Crocosphaera subtropica* ATCC 51142 [199]. The results show that the light-harvesting-balance between photosystem I and II impacts the growth rate and metabolic organization [197].

#### 3.2.3 Richelia

*Richelia* is an obligate symbiont [200] (Fig. 5), having a similar appearance as *Anabaena* with vegetative cells for photosynthesis and heterocysts for N_2_ fixation [201]. Like *Anabaena, Richelia* has heterocysts for N_2_ fixation [31], [202], [203], [204], [205], [206]. *Richelia* is associated with diatoms, providing fixed N to the host diatom [207]; the symbiosis is generally termed a Diatom-Diazotroph-Association or DDA [2], [31], [108]. DDAs have long been recognized [208], [209], and resolved in ecological simulations [104], [106], [108], [190]. Simple equations have been applied to represent DDAs in ocean models, with growth limitation by silica (which is used for diatom’s frustules [104], [106]) and maximum growth rates higher than other N_2_ fixers but lower than non-N_2_ fixers [104], [106]. Using such a trait-based approach a recent modeling study argued that seasonal variations in resource availability would select for faster-growing DDAs in the summer months in the North Pacific Subtropical Gyre, consistent with observations [108]. The hypothesized fast high growth rate of DDAs could be explained by C transfer from the host by a more recently developed coarse-grained model focusing on C and N metabolisms, which also suggests C transfer from the host diatom to *Richelia* to support the high rate of N_2_ fixation [190].

## 4 Resolved elements in coarse-grained models

**Fig. 6:**
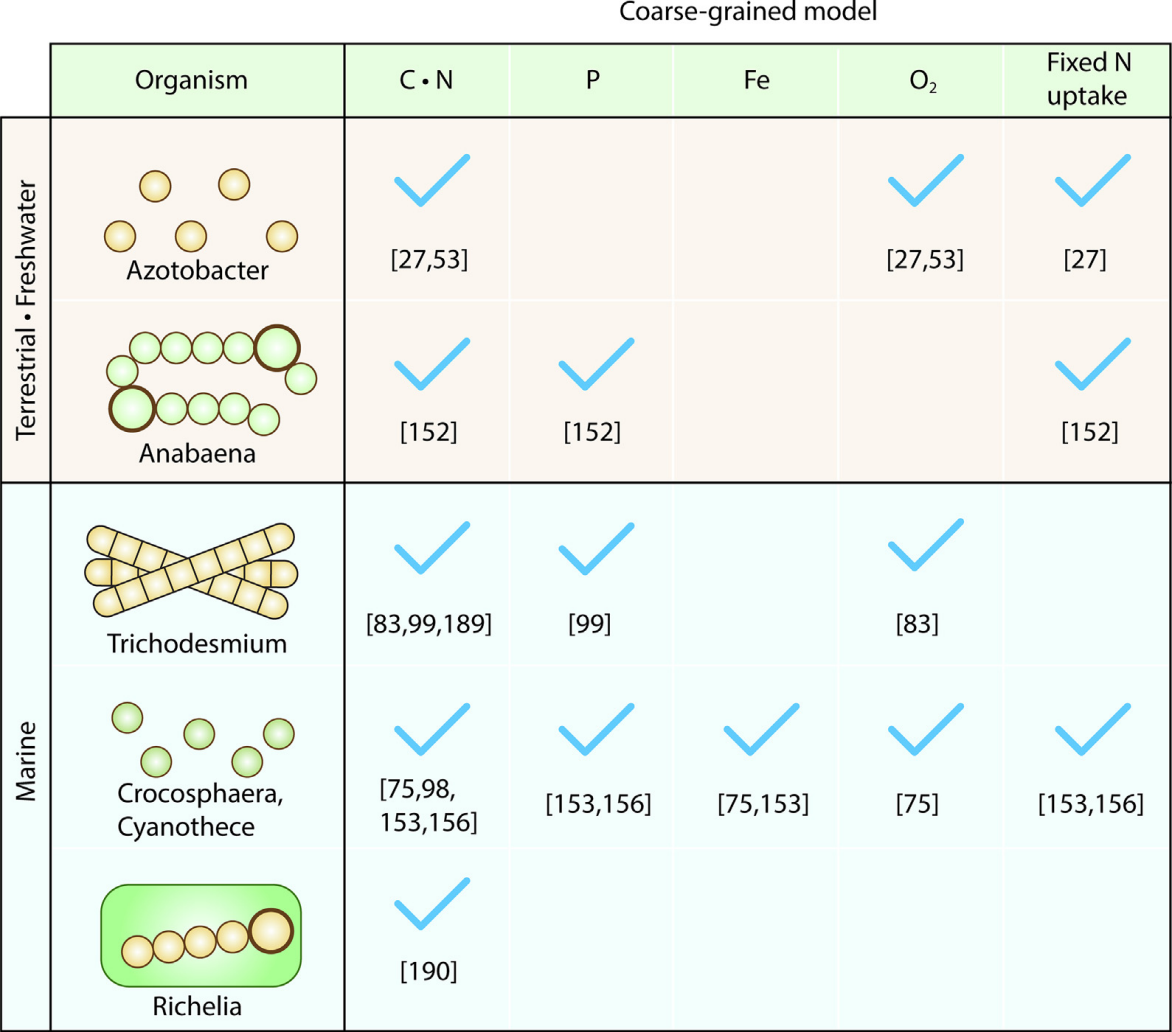
Nitrogen fixers modeled by coarse-grained models and resolved elements. Checkmarks indicate that each element/parameter is simulated. O_2_ indicates intracellular O_2_ and fixed-N uptake indicates uptake of NH_4_^+^ or NO_3_^−^. Numbers below the check marks are example references.

Whereas simple equations and detailed-metabolic models have common forms [100], [104], [106], [188], [190], coarse-grained models are highly variable due to their flexibility to adapt to different purposes [27], [75], [83], [99], [152], [153], [156], [189], [190]. One of the key variations is the number and variety of elements resolved in the models. Many models resolve C and N fluxes but fewer models consider P, Fe (Fig. 6) or other elements explicitly. In this section, we review the variation in coarse-grained models based on an elemental (N, P, Fe) and molecular perspective (e.g., O_2_, NH_4_^+^ and NO_3_^−^ (nitrate)) (Fig. 6) since these resources are known to strongly affect the rate of N_2_ fixation [25], [54], [162], [210], [211], [212], [213].

### 4.1 C and N fluxes

C and N fluxes are key elements in simulating N_2_ fixers since these are major cellular elements [155], [214], [215]. For heterotrophs, fixed C is acquired from the external environment, whereas for autotrophs, they can use CO_2_. C and N are two of the most abundant elements in cells and often growth limiting factors [161], [163], [216]. H and O are generally abundant in the environment (from H_2_O) unless it is arid. As such, C and N have been the central currencies for coarse grained models of N_2_ fixers since their inception [27], [53], [75], [152], [153] (Fig. 6).

### 4.2 P fluxes

P (phosphorus) is essential for cellular growth through its role in nucleic acids, ATP, phosphorylation of various molecules, and other purposes [16], [17]. The cellular P level is sometimes quantified in experiments with marine nitrogen fixers [36], [215], [217], [218], [219], but not as often as C and N, possibly due to the difficulty in measurements. Thus, the data are still limited and accordingly, coarse-grained models resolving P fluxes are limited (Fig. 6). However, a chemostat culture study provided cellular P of *Crocosphaera* [215], and coarse-grained model resolving P has been developed accordingly to the data resolving simplified macromolecular allocation [156]. Also, other optimization models for *Crocosphaera* [153] and *Trichodesmium* [99] resolve P fluxes.

### 4.3 Fe fluxes

Fe is mainly used in photosystems, respiratory complexes, and nitrogenase [56], [220]. Thus, it is essential in cellular growth and maintenance despite the fact that the cellular quota of Fe is small relative to C, N and P [221]. Trace metal measurements require particularly clean laboratory techniques and data on Fe have been relatively scarce. Just a few models have explicitly resolved iron physiology in nitrogen fixers, including studies of *Crocosphaera* [75], [153] and *Trichodesmium* [195] (Fig. 6). Especially, in *Crocosphaera*, the intracellular Fe cycling is shown to be closely coupled with C and N metabolisms [75]. One optimization model [153] used data of external Fe concentration for various growth data [222], to constrain daily average Fe fluxes. Saito et al. estimated Fe allocation from the protein of Fe contents, showing diurnal cycling of Fe between nitrogenase in *Crocosphaera* [56]. This was reproduced by a coarse-grained model of this organism which illustrated its role in organizing the diurnal cycling of cellular metabolisms [75]. A model of *Trichodesmium* resolved Fe to study the response to ocean acidification, predicting that the negative effect of ocean acidification on N_2_ fixation will be especially severe in Fe-limited regions [195].

### 4.4 Fluxes and intracellular concentration of O_2_

Intracellular O_2_ is a key factor in predicting the rate of N_2_ fixation since it negatively affects the activity of nitrogenase [54], [212]. Despite such importance, the direct measurements of intracellular O_2_ are not feasible and models provide a way to interpret the relationship between oxygen and N_2_ fixation. Recent models have explored the impact of respiration and photosynthesis on O_2_ management by a variety of N_2_ fixers. This approach was recently introduced in a coarse-grained model of *Azotobacter* [27], [53] (Fig. 6). Based on the O_2_ fluxes and the assumption of intracellular anoxia, models predicted the presence of a protective barrier reducing the diffusivity of oxygen across membranes as well as enhanced respiration to control intracellular oxygen, consistent with laboratory studies [53]. A similar approach was applied to *Trichodesmium* [83] and *Crocosphaera* [75], suggesting that they also employ a barrier to the invasion of oxygen. These results are supported by the recent observation that N_2_ fixing marine cyanobacteria encode for hopanoid lipids, which would reduce the membrane diffusivity [223]. Notably, the model of *Crocosphaera* suggests that *Crocosphaera* may only survive in high temperature regions (>20 °C), since at lower temperatures respiration rate drops and intracellular O_2_ increases [75].

### 4.5 Fixed N uptake and its influence on N_2_ fixation

The uptake of fixed N (e.g., NO_3_^−^ and NH_4_^+^) has been observed to down-regulate N_2_ fixation [25], [54], [162], [210], [211], [212], [213] (Note that there are cases that such downregulation does not seem to occur [78], [224], [225], [226]). Whereas extensive studies have revealed mechanisms of down-regulation [227], the quantitative models resolving this effect have been scarce (Fig. 6). A coarse-grained model of *Anabaena* resolved the growth based on various fixed N species and the process of their assimilation into biomass. The model captured the observed negative correlation between NO_3_^−^ and NH_4_^+^ uptake and *NifH* (nitrogenase iron protein) level as well as the inhibition of heterocyst differentiation by fixed N [152]. Recently, a coarse-grained model of *Azotobacter* resolved fixed N uptake showing that the rate of N_2_ fixation is optimally regulated, so that biomass concentration is maximized [27]. The model suggested that even when entirely growing on fixed N source, this organism still invested in high rates of respiration associated with respiratory protection. Fixed N uptake was included in a coarse-grained model of *Crocosphaera* based on chemostat culture data, which shows that N_2_ fixation may increase their population despite the presence of NH_4_^+^ [156].

## 5 Remaining challenges

**Fig. 7:**
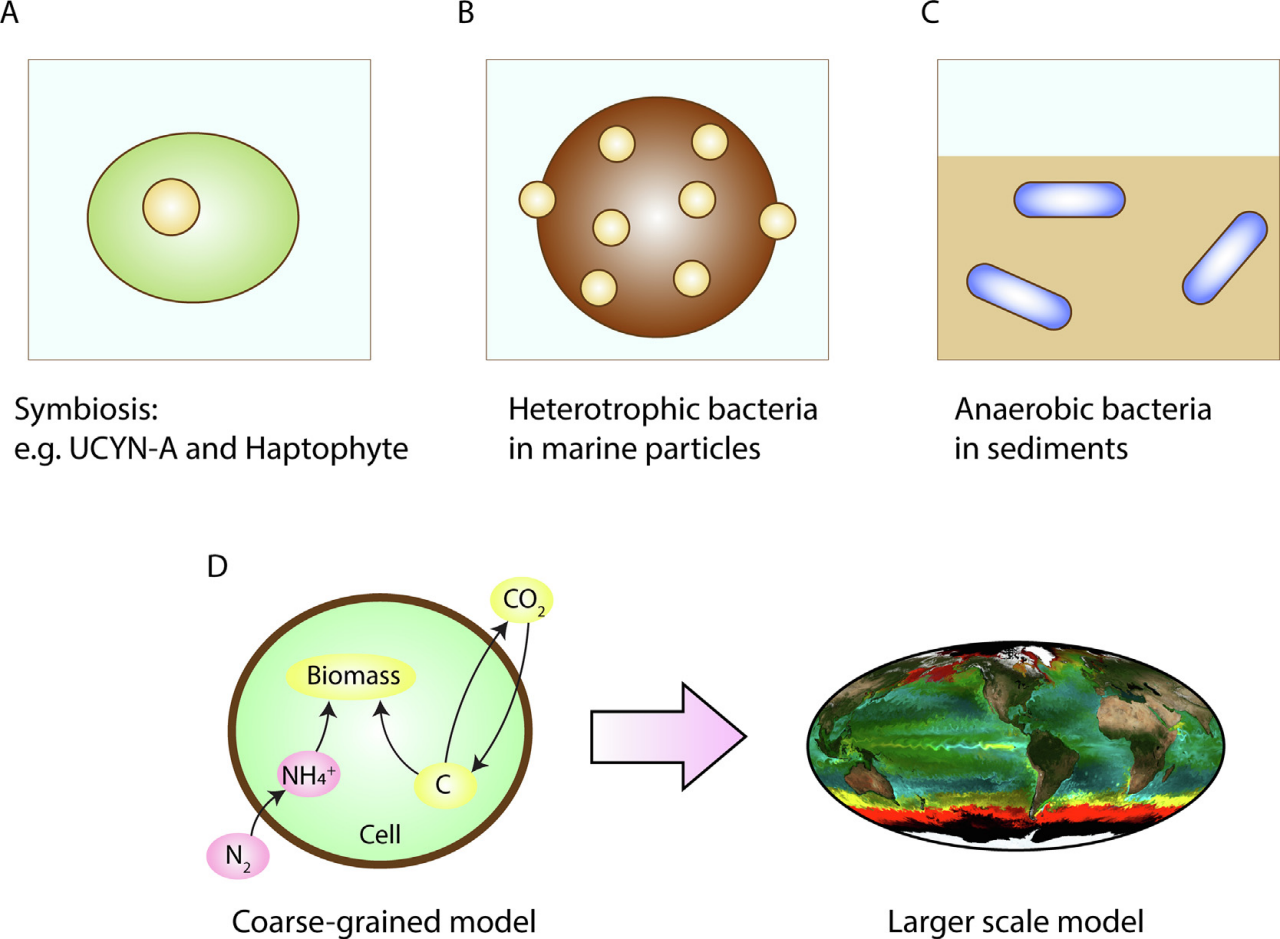
Some future applications of the physiological models of N_2_ fixers. (A)-(C) Organisms that have not been quantitatively modeled. (D) Incorporating coarse-grained models into large-scale simulations. Picture for a large scale model made by Oliver Jahn.

While substantial progress has been made in modeling N_2_ fixers, models have plenty of room to improve in mechanistic and taxonomic breadth and detail (Fig. 7). For example, though relative resource supply and demand may be an important factor in determining the fitness of nitrogen fixers, many coarse-grained models do not resolve key elements (e.g., P, Fe). There are many open questions concerning N_2_ fixation and the physiology of N_2_ fixers [3], [4], [9], [26], [29], [31], [41], [92], [228], [229] and models have a role to play in hypothesizing and testing novel and quantitative explanations. Some important and physiologically interesting N_2_ fixers have not yet been addressed with quantitative models [26], [29]. Here we outline some of the outstanding questions and discuss possible future directions in which modeling contributes to addressing them.

### 5.1 Trichodesmium paradox

*Trichodesmium* fixes N_2_ and photosynthesize during the light period [60], [192]. This is paradoxical since *Trichodesmium* lacks heterocysts and the nitrogenase is sensitive to the O_2_ produced by photosynthesis [54], [212]. The activity of PSII (where O_2_ is produced) switches on and off with a time scale of minutes [92], [230], which would lead nitrogenase to be exposed by O_2_ frequently. A recently developed coarse-grained model resolving average metabolism shows that the residence time of O_2_ is in a time scale of seconds [83]; thus metabolic switching from photosynthesis to non-photosynthesis with high respiration may deplete the intracellular O_2_ quickly. Further modeling to resolve the dynamic regulation of photosynthesis on time scales of minutes may reveal the strategies and associated costs of sustaining N_2_ fixation in the marine environment.

It has been suggested that the microzone of low O_2_ in a colony of *Trichodesmium* plays a role in supporting N_2_ fixation [231]. However, it has been challenged by recent studies that observe higher O_2_ in a colony than the environment [232] and higher N_2_ fixation rates in a free-floating filament than in a colony [84]. Despite that, there are still cases with lower O_2_ in a colony during the middle of the day [84], [233] and models would be useful in exploring the low O_2_ effect as well as why free-floating filaments have higher rates of N_2_ fixation.

### 5.2 Modeling more organisms and outstanding questions

#### 5.2.1 Symbiosis

N_2_ fixers are often found in symbiotic relations [32], [165], [229], [234], [235]. Under N limitation, they provide fixed N to the host supporting their growth. In terrestrial systems, *Rhizobium* and *Anabaena* are well known symbionts with plants [4], [5], [32], [234], but physiological models of these symbiotic relationships are still limited. For example, current models focus mostly on the N_2_ fixers and may not provide a larger picture of symbiosis and nutrient exchanges. How much C should be transferred to the N_2_ fixers for the optimum growth under different conditions? What constrains the rate of N_2_ fixation in symbiosis? Are there ways to increase symbiotic N_2_ fixation by genetic modification? These are still open questions, and models of various levels may provide quantitative predictions and guide empirical studies.

In marine systems, DDA symbioses have long been known [208], [209], but mysteries remain. For example, what molecules do the partners exchange [31], [190]? A recently developed coarse-grained model predicts C transfer from the host diatom leading to the hypothesis that some C molecules are pre-processed within diatoms before transfer to the diazotroph [190]. Simulating N_2_ fixers and hosts together with genome-scale FBA simulations could yield new insight into the types and rates of exchange that would optimize biomass production, which may be tested with laboratory studies [236].

The recently discovered symbiosis between UCYN-A and haptophyte (related to *Braarudosphaera bigelowii*) [29], [228], [237], [238] (Fig. 7A) has been receiving increasing attention. Recent studies show considerable rates of N_2_ fixation and ubiquity of this symbiosis in the global ocean [28], [239], [240], [241], indicating its potential significance in the global N budget and ecosystems. Despite this, theory and models specific to UCYN-A have not been developed, which could provide testable hypotheses addressing outstanding questions such as “what molecules are exchanged?”, “how may such molecular exchange vary under different conditions?”, “how does the symbiotic relationship give an advantage over non-symbiotic N_2_ fixers?” and “why are symbiotic relationships specific?”. Genetic data provide useful qualitative information in modeling the symbiosis. For example, a genetic study revealed a lack of PSII and TCA and Calvin cycles in UCYN-A [242], which can be represented both in coarse-grained models or more detailed metabolic models.

#### 5.2.2 Marine heterotrophic bacteria

More and more genetic studies show that *nifH* gene for heterotrophic bacteria is ubiquitous [26], [243], [244], [245], [246]. However, these studies do not always confirm substantial active N_2_ fixation by these organisms, but such potential has been suggested [26], [247]. What is the contribution to global fixation, why is this functionality so universal, and what are the conditions that allow heterotrophic bacteria to fix N_2_? Marine organic particles (Fig. 7B) have been thought to be loci for N_2_ fixation by these organisms [26], [27], [248], [249]. Particles contain high fixed N, which may suppress N_2_ fixation [25], [210], [211], but would there be a window of time when fixed nitrogen is depleted and N_2_ fixation occurs? Or do they fix N_2_ when the ambient concentration of fixed N is high? Alternatively, respiration in organic particles can provide anoxic microenvironments that circumvent the O_2_ management problem that N_2_ fixers face in the surface ocean [250]. These questions may be quantitatively answered based on a coarse-grained model [27] combined with a simulation of particle environment [251]. In addition to the particles, benthic microbial mats may also provide low O_2_ environment [252], [253], which would also favor N_2_ fixation by heterotrophic bacteria. Physiological model of N_2_ fixers in the context of molecular diffusion in the benthic mat would be useful in quantifying the threshold and the rates for this process.

#### 5.2.3 Anaerobic nitrogen-fixing bacteria

Anaerobic bacteria are also of interest for modeling (Fig. 7C), they mainly exist in sediments or hypersaline environments where O_2_ concentration is low [25], [41]. In such environments, O_2_ is not a major problem for anaerobic N_2_ fixers such as *Clostridium* [41]. How much advantage does the anaerobic environment give to N_2_ fixers? What controls the rate of N_2_ fixation? What mechanisms and conditions allow for N_2_ fixation? In sediments, significant amounts of NH_4_^+^ are detected, but anaerobic N_2_ fixation still seems to occur [25], [41], [210], [211], [254], [255], [256]. Models can help to resolve these questions by quantifying the costs, benefits, and trade-offs of N_2_ fixation in these environments.

### 5.3 Application of coarse-grained models in larger scale simulations

In large scale ecological models, simple equations are used to represent physiologies of N_2_ fixers [101], [104], [106], [107], [114], [129]. However, as for any model, this approach has some limitations. First, such models may not consider the intracellular concentration of O_2_, which can have a significant impact on N_2_ fixation [54], [75]. Second, models generally assume intracellular properties are constant, while in reality they change with the environment (e.g., elemental stoichiometry [85], [215], [218]). Furthermore, these models generally do not consider the effect of fixed N in the environment (e.g., decreased N_2_ fixation due to the presence of NH_4_^+^). One possible solution is to include coarse-grained models into larger-scale models (Fig. 7D). The coarse-grained models lie in a sweet spot between level of detail and computational efficiency and have potential to resolve essential cellular properties [150]. Efforts in this direction have already been started [105], and more modeling tools have been developed (e.g., Cell Flux Models [27], [53], [75], [83]) that can be incorporated in the next generation of ecological models, both for marine and terrestrial systems. Since coarse-grained models require higher numbers of equations and parameters than those of simple equations, constraining them will require continued expansion and curation of accessible laboratory data.

## 6 Enhancing collaboration between theory and observation

**Fig. 8:**
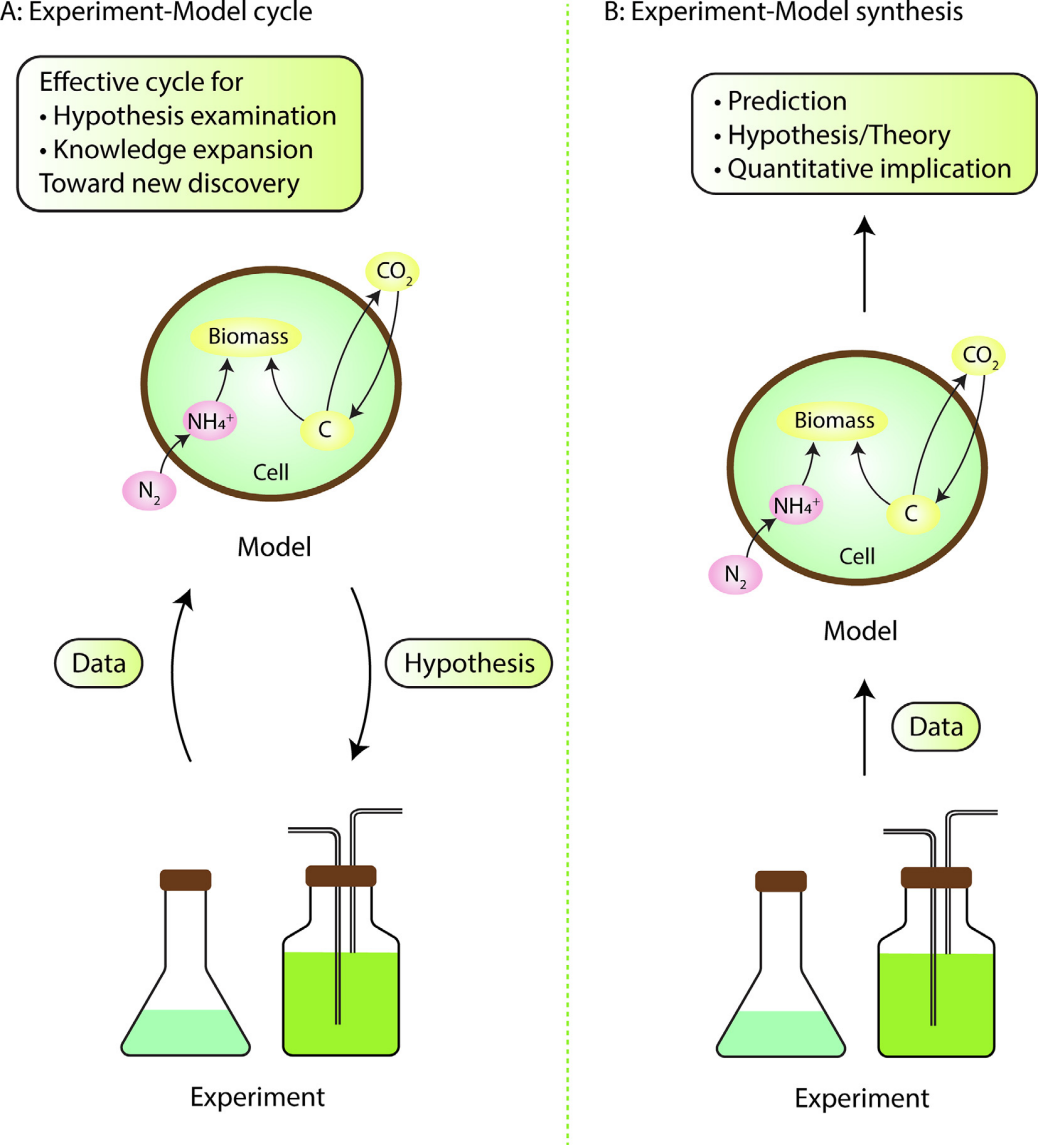
Proposed collaborative schemes between modelers and biologists when studying N_2_ fixation. (A) Model-experiment cycling. (B) Experiment-model synthesis (linear flow). (A) is when model-based hypotheses are testable and (B) is when otherwise. Figure inspired by [257], [258].

**Fig. 9:**
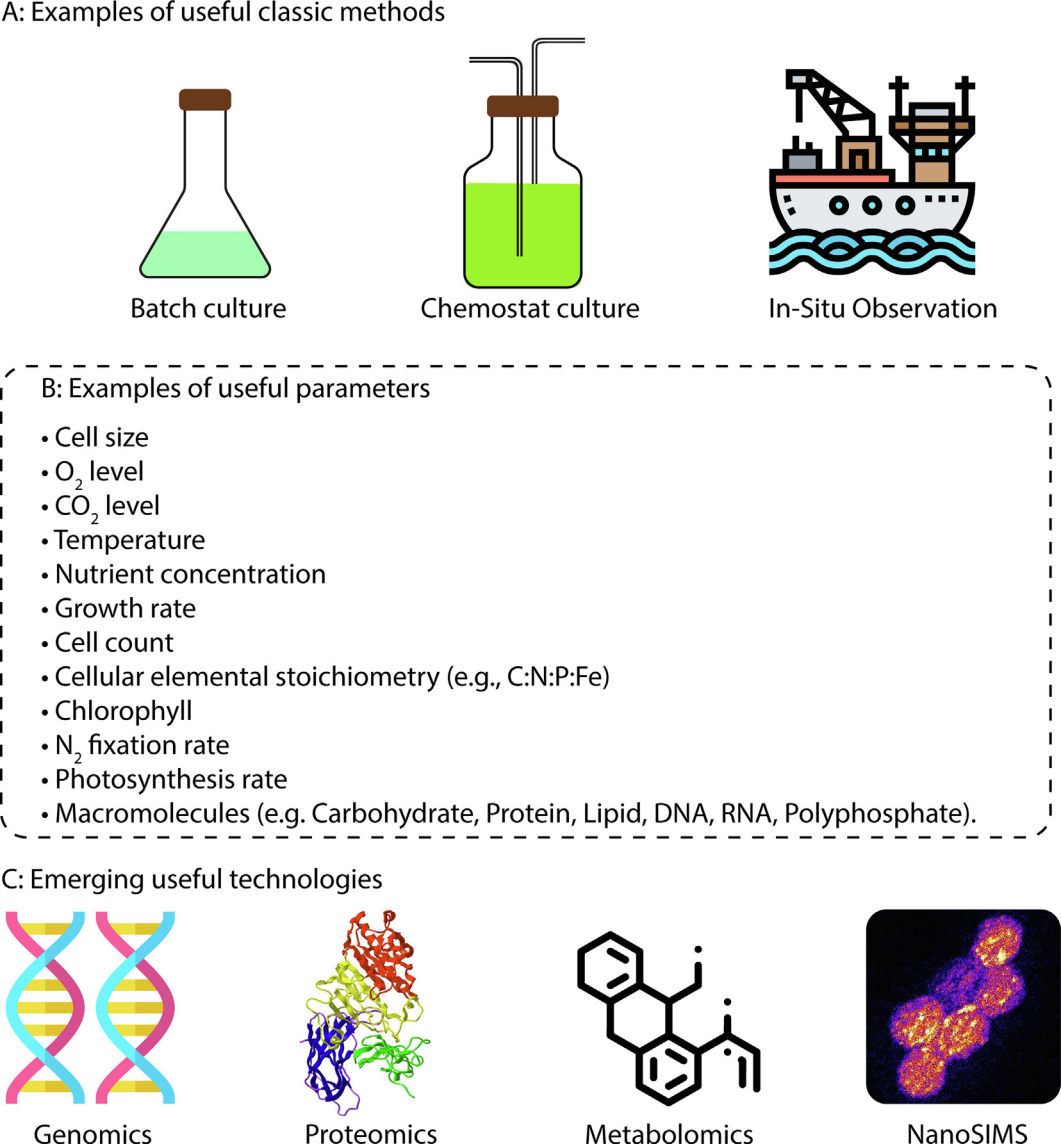
A list of biological experiments and data important for modeling N_2_ fixation. (A) Culturing and sampling methods. (B) List of useful parameters from (A). (C) Emerging technologies that are potentially useful for the models.

Modeling and experiments are complementary to each other (Fig. 8). Experiments are essential in discovering new phenomena and developing conceptual understanding. They provide the quantitative data that is essential for testing theories and constraining parameterizations. Models are often useful for synthesizing and organizing understanding, interpreting observed phenomena, as well as stimulating new hypotheses and testable predictions. An increasing number of studies combine these two different types of approaches, but its considerable potential remains only partly realized. In this section, hoping to stimulate more of such collaborations, we describe two types of model-experiment collaborations (Fig. 8) and list examples of useful data for developing models (Fig. 9).

### 6.1 Experiment-model cycles

One type of collaboration is the experiment-model cycle (Fig. 8A). Experiment provides ingredients for computational models which produce new, testable hypotheses stimulating further experimentation. Also, in time, model predictions can be tested by experimental measurements, which may lead to modification of modeling. This type of cycle was proposed for Systems Biology during the beginning of the 21st century [257], [258] and applies to N_2_ fixers as well. For example, based on laboratory data, coarse-grained models suggested the existence of a strong barrier for O_2_ diffusion [75], [83], which can be experimentally tested by analyzing the properties of cellular membrane. In fact, the supporting evidence has been shown recently with genetics study [223]. Based on the cellular-size information from observation, a coarse-grained model of DDAs suggested the existence of significant C transfer from the host diatom to N_2_ fixer in DDA [190]. This model-derived hypothesis may also be tested, for example, with NanoSIMS experiments (a technique for visualizing spatial patterns of elemental accumulations [28], [191], [259], [260]), which in turn may change model parameterization. This cycle leads to the deep, robust, and mechanistic understanding of the cellular system of N_2_ fixers.

### 6.2 Experiment-model synthesis

Another type of collaboration is a rather simple one-time combination of experiment and model, which provides theory and quantitative implications (Fig. 8B). This can be applied when the model results may not be tested by experiment easily or when technical barriers preclude experimental tests. For example, a recent NanoSIMS study showed heterogeneity in multiple types of unicellular N_2_-fixing cyanobacteria (some cells fix N_2_ and others do not), based on which a coarse-grained model was developed, showing that such heterogeneity reduces C costs and expands the depth niche on N_2_ fixers in the open ocean [191]. This model prediction is hard to test in observation or experiments, since we still do not know how to experimentally modulate the number of active cells. Based on a batch culture study, another coarse-grained model was developed showing that respiration rate drops with temperature, which in turn leads to increase in O_2_ concentration in the cell, reducing the rate of N_2_ fixation [75]. This hypothesis is rather difficult to test, as intracellular O_2_ may not be measured with current techniques. In these cases, models are used to complement experiments, expanding the view/implication based on quantitative theories.

### 6.3 Examples of useful experimental methods

#### 6.3.1 Chemostat culture

Chemostat culture is a widely used method providing essential data for quantitative models (Fig. 9A). Its strength is based on that the steady state is created in the culture where the cellular growth rate is known from the dilution rate (flow rate of the medium) [157], [159], [261]. Since the growth rate and steady state condition are useful factors in constraining all types of models, the data from chemostat culture have been widely used in modeling studies [58], [157], [159], [161], [162], [163], [192], [215], [262], [263], [264] because the steady state makes for mathematically simple and tractable models. In particular, many of the coarse-grained models have been developed based on chemostat data [27], [53], [98], [99], [152], [153], [156]. The method can be labor intensive [159] and technically challenging, limiting the number of available data. However, the method has high value for the development of coarse-grained models.

#### 6.3.2 Batch culture

In batch cultures a nutrient-rich medium is inoculated with live cells whose population grows and consumes the resources [211], [217], [265], [266], [267] (Fig. 9A). Over time, the nutrients are depleted and population growth slows. The strength of this method is its simplicity relative to the chemostat culture. The environment within the culture changes continuously, so time-dependent models are required to simulate and interpret these experiments. However, for models built on a dynamical framework that captures time-dependent biological responses [75], [99], [152], [153], the batch culture data can be of great use. If acclimation occurs sufficiently rapidly that cellular composition stays close to optimal over the time-course of the experiment, we might use a quasi-steady state modeling approach to represent the physiology. There have been efforts to adapt FBA to dynamic situations [147], [148], [268] and this approach has started to be applied to N_2_ fixers [149].

#### 6.3.3 Observation (field measurements)

Field observations and *in situ* measurements (Fig. 9A) are highly valuable for modeling. However, the environment is highly complex and often challenging to use such data for model parameterization for individual organisms. For example, in the ocean, microbial populations are very diverse and mixed. However, combinations of technologies such as meta-‘omics’, [269], [270], [271], [272], [273], [274], [275] flow cytometry [225], [238], [276], FISH (Fluorescent *In Situ* Hybridization) [28], [225], [238], [277] and NanoSIMS [28], [207], [225], [259], [260] allow observation and parametrization down to the level of individual cells. Surveys of biogeochemical fluxes including N_2_ fixation can be compiled for comparison with larger-scale ocean and terrestrial ecosystem simulations [101], [102], [104], [106]. Global coverage of rates of N_2_ fixation is still sparse [88], [89], [278], but recent technological development allows high-frequency measurements of N_2_ fixation [86], [279], allowing for rapidly increasing data coverage over time and space scales of the ocean.

### 6.4 Examples of useful parameters

Models can help select and prioritize the key parameters for which laboratory studies and field observations are most needed to resolve outstanding questions, as illustrated in Fig. 9B. Cell size provides hints for diffusivity of O_2_ into the cell [53], [66], [83], [84] as well as approximates cellular compositions [280], [281], [282]. To quantify O_2_ fluxes and intracellular O_2_, data on O_2_ concentrations in the culture/environment are useful [61], [84], [232]. CO_2_ level is also important for photosynthetic organisms as it may affect the rate of photosynthesis and thus O_2_ evolution [35], [283]. Unless testing the effect of CO_2_ limitation, it is preferred that CO_2_ is pumped in the culture to avoid the negative effect of CO_2_ limitation on photosynthesis, as such effect would make the model parameterization complex. Temperature is another important factor as it affects the molecular diffusion [284], [285] and cellular metabolisms [286], [287], [288]. Growth rate is a known parameter for chemostat cultures [157], [159], [261], but it is also important for batch cultures, since many model outputs are related to growth rates (e.g., N_2_ fixation, respiration, photosynthesis, elemental stoichiometry [158], [161], [215], [264], [289], [290]). Cell concentration is required if it is necessary to obtain per cell values such as elemental or molecular mass. Cellular elemental stoichiometry provides the cellular demand for each nutrient for a specific growth rate [58], [215], [218]. It is known to vary with growth rate, thus, values for multiple growth rates are ideal (preferably at least 3 growth rates in case the relation is non-linear) [158], [215], [291]. For photosynthetic N_2_ fixers (e.g., *Anabaena, Crocosphaera, Trichodesmium*), the photosynthesis-related parameters such as cellular content of chlorophyll [215], [264] and the rate of photosynthesis [85], [192], [287] are useful as photosynthesis produces fixed C essential for cellular growth and metabolisms as well as O_2_, which is detrimental to N_2_ fixation. The rate of N_2_ fixation is the essence of N_2_ fixers and certainly is useful. More recent models include macromolecular allocations [121], [156], [191] and related data, such as the levels of lipid, carbohydrate, chlorophyll, protein and nucleic acids [123], [144], [292] are useful in testing the model output from these types models. Different studies use different units for output data: some use per chlorophyll [192], [219], [293], [294], other use per C or N [35], [213], [262], per cell [58], [85], [264], [295], per cellular volume [215] or per cell suspension volume (e.g., seawater) [218]. Ideally, these units are inter-convertible and, for this, the values for chlorophyll per cell, C and N per cell, and cellular concentration are valuable. Especially, chlorophyll content is highly variable [158], [215], [264], [296], [297] and the data for chlorophyll (per cell or per C) would be of great use if the data are to be presented per chlorophyll.

### 6.5 Emerging experimental methods and data

Technological and experimental advancements provide new types of data available for model development (Fig. 9C). Proteomics and genomics indicate the presence of metabolic pathways, which provide a basis for FBA [100], [188]; FBA predicts a metabolic flux network (and thus the partition of fluxes at metabolic branch-points) based on possible sets of reactions informed from these ‘omics studies and the flux optimization for selected purposes (e.g., maximizing biomass production) [100], [137], [138], [149], [188]. The information from genomics can also be useful for coarse-grained models, since the model can selectively reflect distinct metabolic patterns [242]. Proteomics can reveal the allocation to enzymes that mediate key functions such as N_2_ fixation and photosynthesis [56], which have been resolved in some models [75], [99], [152], [153], [186]. Also, some coarse-grained models coarsely resolve protein allocation and could be better constrained with more proteomics data. In the future, the rapidly advancing capability to measure the presence and relative abundance of metabolites, known as metabolomics [298], [299], may complement FBA models, together leading to quantification of both metabolites and metabolic fluxes.

Sitting in between genomics and proteomics is transcriptomics, providing the quantitative information for the level of specific mRNAs [271], [274], [275]. Since a large part of mRNAs are used for protein synthesis, transcriptomics provides implication for what proteins are expressed/used within the cell. This measurement may not strictly predict the level of proteins, since it does not provide information for the destruction of proteins (e.g., protein turnover [300]). Despite that, this technology has been widely used due to low cost and low time requirement relative to proteomics.

Furthermore, metabolomics may be used to approximate the composition of macromolecules, which would be useful in constraining coarse-grained models that resolve macromolecular allocations. For example, comprehensive measurements of cellular amino acids [301] may be useful in estimating the level of cellular proteins. Finally, NanoSIMS technology provides useful data in elemental accumulation at (sub)cellular levels [28], [191], [259], [260], essential in modeling heterogeneous cellular activities [191], providing another layer of detail in modeling at any scale.

## 7 Summary and outlook

Overall, each type of model - simple equations, coarse-grained, and detailed metabolic models - has its own strength and can be applied to different problems. The coarse-grained type has been applied to a wide range of applications and provided many new insights, and still holds potential for further development. Proper experimental data are essential for any type of modeling, and both classic parameters and more recent technologies provide useful information. Experiments and models are complementary and provide powerful synthesis of quantitative measurements and theory. This synthetic approach has been rapidly expanding. With such model-experiment synthesis, models can be expanded to cover different diazotrophic organisms, such as UCYN-A, marine heterotrophic N_2_-fixers, and anaerobic N_2_ fixers. As the emerging class of coarse-grained models are incorporated into large-scale models, we expect a rapid development and expansion of predictive skill and understanding of the interactions between microbial ecosystems, biogeochemistry, and climate.

## Author contributions

K.I. wrote the original draft, which was reviewed and edited by all the co-authors. The project was administered by K.I. and T.M. and supervised by C.D., O.P. and M.J.F. All the co-authors contributed to funding acquisition.

## Declaration of Competing Interest

The authors declare that they have no known competing financial interests or personal relationships that could have appeared to influence the work reported in this paper.
